# Human MSCs promotes colorectal cancer epithelial–mesenchymal transition and progression via CCL5/*β*-catenin/Slug pathway

**DOI:** 10.1038/cddis.2017.138

**Published:** 2017-05-25

**Authors:** Ke Chen, Qianqian Liu, Lai Ling Tsang, Qiao Ye, Hsiao Chang Chan, Yunwei Sun, Xiaohua Jiang

**Affiliations:** 1 Department of Gastroenterology, Rui-jin Hospital, Shanghai Jiao Tong University School of Medicine, Shanghai, China; 2 Key Laboratory for Regenerative Medicine, Ministry of Education, Epithelial Cell Biology Research Center, Faculty of Medicine, School of Biomedical Sciences, The Chinese University of Hong Kong, Hong Kong SAR, China; 3 School of Biomedical Sciences Core Laboratory, the Chinese University of Hong Kong Shenzhen Research Institute, Shenzhen 518057, China

## Abstract

**Mesenchymal stem cells (MSCs) extensively interact with cancer cells and other stroma cells in the tumor microenvironment. However, the role of MSCs in colorectal cancer (CRC) progression and metastasis is controversial. This study was designed to identify the role of inflammation-activated-MSCs in CRC development. Our results show that tumor necrosis factor (TNF)-*****α*****-preactivated-hMSCs significantly promote the progression of colon cancer cells by enhancing cell proliferation, epithelial–mesenchymal transition, migration, and invasion. TNF-*****α*****-primed-hMSCs secrete high level of CCL5, which interacts with its receptor CCR1 expressed in colon cancer cells. Interestingly, the stimulation of colon cancer cell progression by TNF-*****α***-**primed hMSCs is associated with the upregulation of** ***β*****-catenin signaling pathway. Blocking** ***β*****-catenin pathway significantly decreases the TNF-*****α*****-primed-conditioned medium or CCL5-mediated cancer cell progression by decreasing the enhancement of Slug, suggesting that the CCL5/*****β*****-catenin/Slug pathway plays a critical role in hMSC-mediated cancer progression. Furthermore,** ***in vivo*** **model in nude mice confirms the ability of hMSCs to promote the proliferation and progression of colon cancer cells, and the upregulation of CCl5/*****β*****-catenin/Slug pathway. Taken together, the present study has demonstrated a novel pathway involving CCl5/CCR1/*****β*****-catenin/Slug, via which hMSCs promotes CRC development.**

Colorectal cancer (CRC) is the third most common cancer and the third leading cause of cancer-related death worldwide.^1^ Metastatic spread is one of the hallmarks of CRC and is the major cause of patient death.^2^ Indeed, the 5-year survival rate of patients dramatically declines from ~90% in early stage non-metastatic tumors to ~5% in cases with distant metastasis.^3^ Beyond the intrinsic genetic lesions that affect the CRC cells, a dynamic interaction occurs between cancer cells and the host stromal microenvironment to support cancerous growth and dissemination.^4^ The CRC stroma, which is comprised of immune cells, fibroblasts, extracellular matrix constituents, growth factors, and cytokines/chemokines, plays an essential role in tumor progression and metastasis, and referred to as a “ reactive stroma”.^5, 6^

MSCs are one of the major components of the tumor stroma, which are believed to be the precursors of tumor-associated fibroblasts.^7, 8, 9, 10^ Tumor-resident MSCs are often constantly exposed to immune cells and inflammatory cytokines/chemokines in the tumor microenvironment.^5^ In turn, they may acquire functions that are distinct from normal tissue MSCs, which subsequently modulate the tumor microenvironment and ultimately affect tumor progression.^11^ Previous studies on the role of MSCs in CRC development have yielded conflicting results, some reports showed that MSCs inhibited tumor growth whereas others demonstrated that MSCs promoted the initiation and development of CRC.^12, 13, 14, 15, 16, 17^ For instances, it was reported that MSCs inhibited AOM-induced tumor initiation by preventing the initiating cells from sustaining DNA insults and subsequent G_1_ arrest in mice.^13^ In contrast, De Boeck *et al.* demonstrated that MSCs promoted the invasion, survival, and tumorigenicity of CRC cells both *in vitro* and *in vivo* through paracrine neuregulin 1/HER3 signaling.^15^ The seemingly contradictory findings indicate that the role of MSCs in CRC development might be stage-dependent and microenvironment-dependent. On the other hand, the detailed mechanisms through which MSCs acquire their tumor suppressing/promoting function during CRC development are largely unknown.

CCL5/RANTES is one of the C–C chemokines secreted by various cell types including platelets, immune cells, fibroblasts, endothelial, and epithelial cells, which interacts with the G-protein-coupled receptors CCR1, CCR3, and CCR5.^18^ Although CCL5 has been originally identified as an inducer that recruits leukocytes to sites of inflammation,^19^ accumulating evidence has clearly shown that CCL5 is highly expressed in various tumors. CCL5 has been demonstrated to promote tumor development and metastasis by inducing tumor cell proliferation, angiogenesis, or expression of matrix metalloproteinases.^20, 21, 22, 23^ Of note, recent studies have shown that CCL5 plays a critical role in CRC development.^22, 24, 25^ Patients with high CCL5 levels have been observed to have poorer prognosis and higher resistance to anti-cancer drugs than patients with low CCL5 levels.^22, 26^ In addition, CCL5 increases the *in vitro* growth and the migratory responses of CRC cells from both human and mouse origins.^24^ More interestingly, CCL5 has been demonstrated to be an important factor responsible for immune escape in cancer by increasing the accumulation of myeloid-derived suppressor cells and T-regulatory cells during the development of CRC,^27, 28^ indicating that CCL5 is important for mediating regulatory effects in CRC development through the interaction of stroma cells and cancer cells. On the other hand, it has been reported recently that MSCs secret CCL5, which is critical for maintaining the MSCs identity and multi-potency.^29^ In addition, CCL5/CCR1 axis is pivotal for the communication between MSCs and their target tissues.^30, 31^ Altogether, these findings make us to hypothesize that CCL5 may play a role in mediating a synergistic crosstalk between MSCs and cancer cells to sustain CRC growth and metastasis.

We undertook the present study to determine the role of human MSCs on CRC development both *in vitro* and *in vivo*. Our results show that tumor necrosis factor (TNF)-*α* preactivated-hMSCs secrete high levels of CCL5 and promote CRC progression. The tumor-promoting effect of MSCs is attributed to the activation of epithelial–mesenchymal transition (EMT) process, which is mediated by CCL5/CCR1/*β*-catenin/Slug pathway.

## Results

### TNF-*α* aggravates the promotive effect of hMSCs on colon cancer cell proliferation

**Figure 1:**
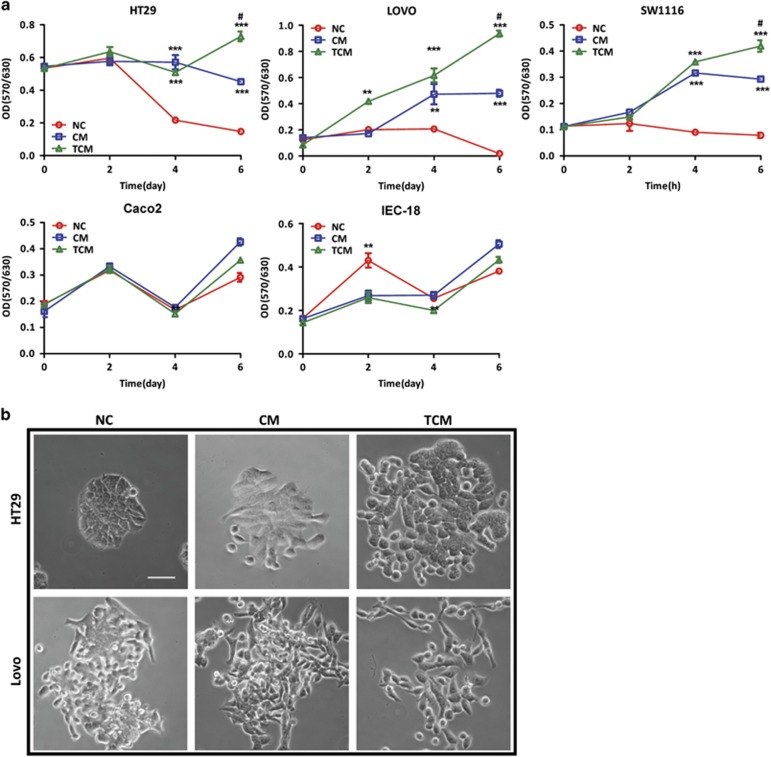
TNF-*α* aggravates the promotive effect of hMSCs on colon cancer cell proliferation. (**a**) Conditioned media from hMSCs promotes the proliferation of CRC cell lines. HT29, Lovo, Caco2, and IEC-18 cells were cultured in the CM/TCM collected from hMSCs or serum-free media (NC) for 6 days, then cell proliferation was assessed using the MTT assay. The experimental procedure was repeated for three times, **P<0.01 *versus* control, ***P<0.001 *versus* control, ^#^*P*<0.05 *versus* hMSCs; (**b**) Effects of hMSC-CM/TCM on morphological change of HT29 and Lovo cells after cocultured with untreated hMSCs or TNF-*α*-treated-hMSCs for 4 days (scale bar, 500 *μ*m)

As tumor-resident MSCs are often constantly exposed to inflammatory cytokines, we reasoned that they might acquire distinctive functions on cancer development compared to normal tissue MSCs. To test this hypothesis, we first examined the effect of conditional medium collected from inactivated or TNF-*α*-activated hMSCs (conditional media (CM)/TCM) on colon cancer cell growth. Human colon cancer cell lines (HT29, Lovo, SW1116, and Caco2) or normal intestinal epithelial cell line IEC-18 were cultured with hMSC-derived CM/TCM or serum-free media (NC) for consecutive 6 days, and their proliferative capacity was determined by MTT assay. Our results showed that while CM significantly promoted cell proliferation in HT29, Lovo and SW1116, the effect of TCM was much more pronounced at day 6 after treatment. In contrast, neither CM nor TCM had any promotive effect on well-differentiated colon cancer cell line Caco2 or normal intestinal epithelial cell line IEC-18 (Figure 1a). These results indicate that hMSCs promote cell growth in lower-differentiated colon cancer cells, and this effect is more prominent with TNF-*α* pretreatment.

### Preactivated-hMSCs promote metastatic phenotype of colon cancer cells

**Figure 2:**
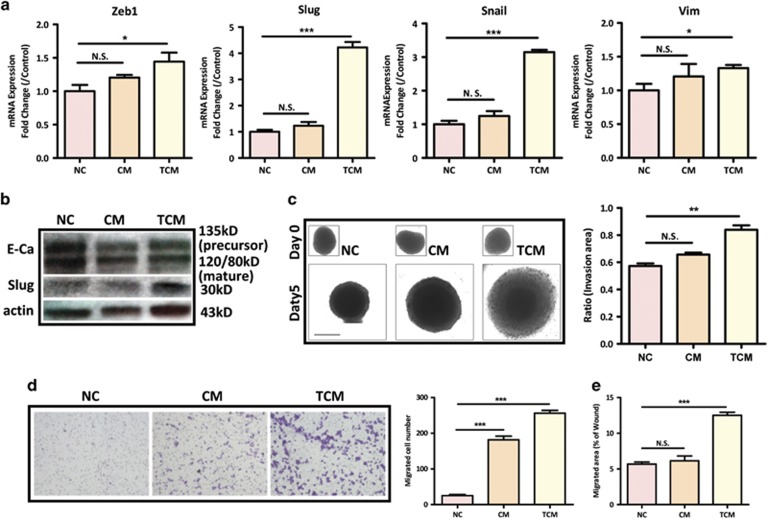
hMSCs promote metastatic phenotype of colon cancer cells. (**a**) After incubation with CM or TCM, the expression levels of EMT-related genes in HT29 were evaluated by quantitative PCR. Data are presented as the means±S.D. *n*=3. NS, no significance. *P<0.05, ***P<0.001 *versus* control; (**b**) Western blot analysis showed that CM and TCM decreased the expression of E-cadherin, whereas TCM increased the expression of Slug; (**c**) Invasion ability of HT29 treated with CM or TCM was evaluated by 3D spheroid invasion assay (scale bar, 500 *μ*m). Invasion ratio=((area D5−area D0))/(area D0). The experiment was repeated three times, **P<0.01 *versus* control group; (**d**) Cell migration was determined by transwell assay in SW1116. 1 × 10^4^ SW1116 cells were seeded in the upper chamber whereas CM or TCM were administrated in the lower chamber. The experiment was repeated three times. ***P<0.001 *versus* control group; (**e**) wound healing assay was used to determine cell migration. Quantification data was presented as mean±S.D. from three independent experiments, ***P<0.001 *versus* control group

In colon cancer, acquisition of a mesenchymal-like phenotype, that is reminiscent of an EMT, is associated with pro-metastatic properties including increased migration, invasion, and cancer stem cell characteristics.^32^ To further understand whether hMSCs contribute to EMT process and colon cancer progression, we cocultured HT29 or Lovo cells with untreated hMSCs or TNF-*α*-pretreated hMSCs via transwell for 4 days, and determined their morphological and molecular changes related to EMT. Our results showed that after co-incubation with hMSCs, both HT29 and Lovo acquired a pronounced transition from a typical round/oval and clustered morphology to a more elongated and scattered morphology, which is indicative of EMT process. Of note, this morphological change was more evident in preactivated-hMSCs group (Figure 1b). Next, we examined the expression of key transcription factors governing EMT process (*Zeb1*, *Snail*, and *Slug*) and mesenchymal marker Vimentin (*Vim*) in HT29 cocultured with CM or TCM. Our real-time PCR results showed that while CM slightly increased the mRNA expression of EMT markers, TCM dramatically upregulated the expression levels of key transcription factors, especially *Snail* and *Slug* in HT29 (Figure 2a). Consistently, our western blot results demonstrated that TCM significantly decreased the expression of E-cadherin, but increased the expression of Slug in HT29 (Figure 2b). To further examine the effect of hMSCs on EMT-associated phenotypes, we proceeded to evaluate the migratory and invasive abilities of colon cancer cells treated with CM or TCM. Since HT29 cells showed limited migratory ability in transwell assay, a 3D spheroid invasion analysis was applied. While HT29 spheroids embedded in Matrigel did not develop invasive properties, TCM treatment dramatically induced HT29 invasion into the surrounding matrix (Figure 2c). Moreover, a more invasive colon cancer cell line SW1116 was used for the wound healing and transwell migration assay. As shown in Figures 2d and e, while both CM and TCM promoted the migratory ability of SW1116 in transwell migration assay, only TCM significantly stimulated migration in would healing assay. In addition, TCM-induced EMT markers more significantly in SW1116 (Supplementary Figure 1). Taken together, these results indicate that preactivated-hMSCs promote an EMT phenotype with enhanced metastatic capability in colon cancer cells.

### CCL5 plays an important role in hMSC-mediated colon cancer progression

**Figure 3:**
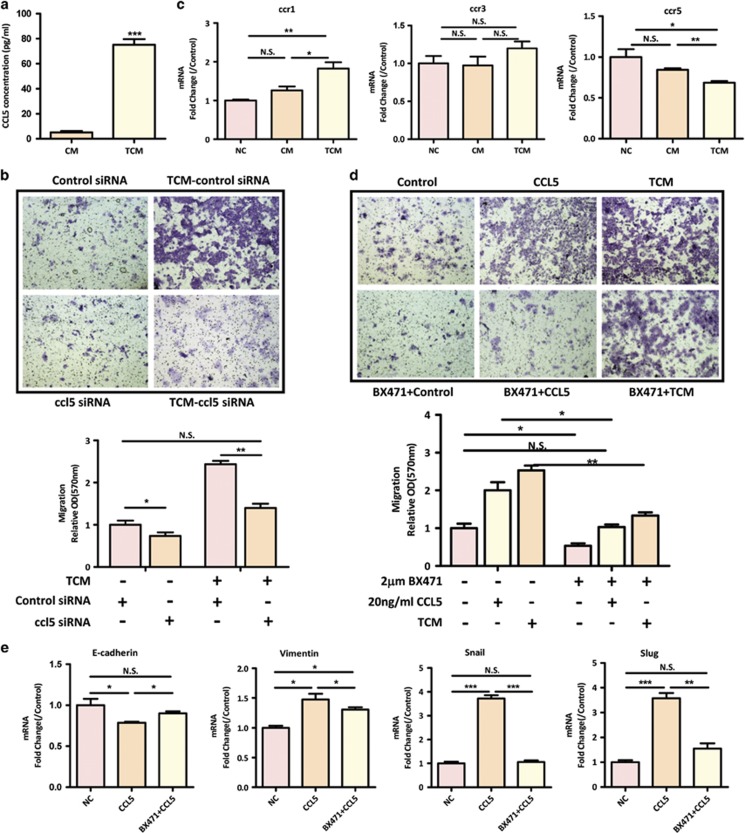
CCL5/CCR1 axis mediates hMSC-enhanced metastatic phenotype. (**a**) The concentration of CCL5 in CM or TCM was determined by ELISA assay from three independent experiments. ***P<0.001 *versus* control group; (**b**) after 24 h of scrambled or ccl5 siRNA treatment, hMSCs were treated with 10 ng/ml TNF-*α* for 24 h and collected for TCM. SW1116 cells were plated in the upper chamber and evaluated their migratory ability toward TCM by transwell assay. Quantification was presented as mean±S.D. from three independent experiments. *P<0.05; ** *P*<0.01, NS, no significance; (**c**) real-time PCR analysis of ccr expression in SW1116 cells treated with CM or TCM for 24 h. Data are presented as the means±S.D. *n*=3. *P<0.05; **P<0.01; (**d**) The effect of CCR1 antagonist BX471 on TCM- or CCL5-induced migration was determined by transwell assay. 2 *μ*M BX471 was added into SW1116 cells and seeded in the upper chamber, TCM or 20 ng/ml CCL5 were added in the lower chamber. Quantification was presented as mean±S.D. from three independent experiments.*P<0.05,**P<0.01; (**e**) 2 *μ*M BX471 was added simultaneously with 20 ng/ml CCL5 into SW1116, the expression of EMT markers was determined by real-time PCR. Data are presented as the means±S.D., *n*=3. *P<0.05, **P<0.01, ***P<0.001

Given that only activated-hMSCs promote colon cancer progression but not inactivated hMSCs, we focused on TNF-*α* preactivated-hMSCs in the following mechanistic study. As CCL5 has been implicated in the interaction between stromal cells and colon cancer cells,^27^ we first compared the expression levels of CCL5 in the CM or TCM derived from hMSCs. As shown in Figure 3a, the CCL5 secretion had a 14.7-fold increase in TNF-*α*-stimulated hMSCs compared to unstimulated hMSCs as determined by enzyme-linked immunosorbent assay (ELISA). To validate the causative role of paracrine CCL5 in colon cancer progression, we took loss of function approach by using ccl5 siRNAs. For ccl5 knockdown experiments, hMSCs were transfected with ccl5 siRNA or control siRNA, and then treated with TNF-*α*. We were able to significantly reduce the mRNA expression and secretion of CCL5 in untreated and TNF-*α* pretreated hMSCs (Supplementary Figures 2a and b). We then collected TCM from either control siRNA-treated or ccl5siRNA-treated hMSCs and determined the migratory capacity of SW1116 toward the TCM. Strikingly, ccl5 siRNA transfection completely abolished the stimulatory effect of TCM on SW1116 migration (Figure 3b), indicating that paracrine secretion of CCL5 is essential for the effect of TCM. To further determine which receptor on colon cancer cells is responsible for the paracrine effect of CCL5 secreted by hMSCs, we examined the mRNA expression levels of ccr1, ccr3, and ccr5 in SW1116. Interestingly, while all three CCL5 receptors could be detected in SW1116, only ccr1 was found to be significantly upregulated with TCM treatment, which is in parallel with CCL5 upregulation (Figure 3c). The effects of CCR1 on the migration and EMT were assessed by using CCR1 specific inhibitor BX471 in SW1116 cells. To do this, SW1116 was pretreated with BX471 and added in the upper chamber, whereas TCM or CCL5 were administrated in the lower chamber. Our results showed that BX471 significantly attenuated the migratory capability of SW1116 toward TCM or CCL5 (Figure 3d). In addition, suppression of CCR1 also alleviated CCL5-stimulated EMT, as supported by the upregulation of *E-cadherin*, and down-regulation of *Vimentin*, *Slug* and *Snail* (Figure 3e). Collectively, these results indicate that CCL5/CCR1 axis plays an important role in preactivated-hMSC-mediated colon cancer progression.

### CCL5 activates *β*-catenin/Slug pathway in colon cancer cells

**Figure 4:**
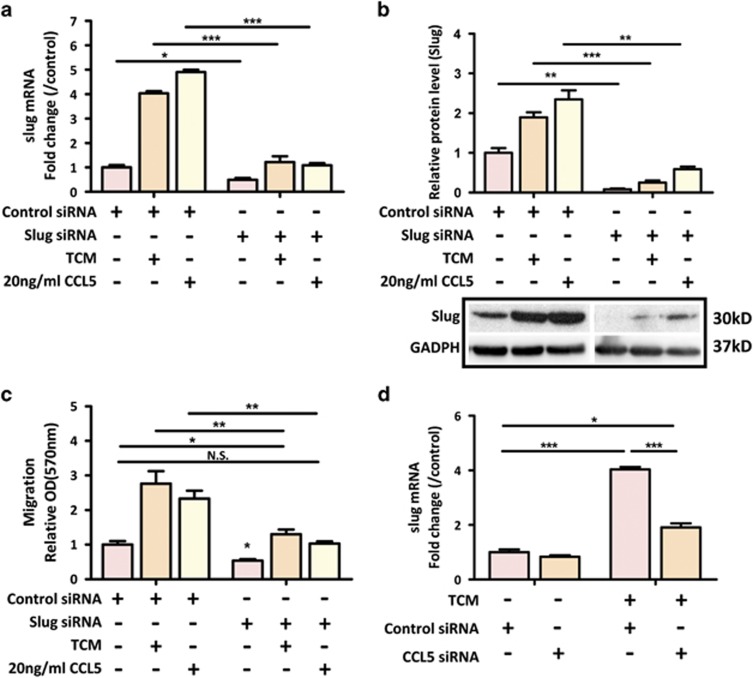
CCL5 activates Slug in colon cancer cells. (**a**) After Scrambled or Slug siRNA treatment, the SW1116 cells were exposed to TCM or 20 ng/ml for 24 h. The expression of Slug was determined by real-time PCR analysis in SW1116. Data are presented as the means±S.D. *n*=3. *P<0.05, ***P<0.001; (**b**) The expression of Slug was determined by western blot analysis in SW1116 treated with TCM or 20 ng/ml CCL5 for 24 h. Data are presented as the means±S.D. *n*=3. **P<0.01, ***P<0.001. Representative blot was shown below; (**c**) After 24 hours of Scrambled or Slug siRNA treatment, SW1116 cells were seeded in the upper chamber and examined their migratory capability to TCM or CCL5. Quantification was presented as mean±S.D. from three independent experiments. *P<0.05; **P<0.01; (**d**) After 24 h of Scrambled or ccl5 siRNA treatment, hMSCs were treated with 10 ng/ml TNF-*α* for 24 h and collected for TCM. SW1116 cells were treated with TCM for 24 h and examined for Slug expression by real-time PCR. Data are presented as the means±S.D. *n*=3. *P<0.05, ***P<0.001

Till now, the relationship between paracrine effect of CCL5 and inherent EMT signaling in cancer cells has been poorly characterized. Given that Slug is the most upregulated EMT-related transcriptional factor in response to TCM (Figures 2a and b), we sought to test the hypothesis that Slug might be involved in CCL5-mediated colon cancer progression. We first knocked down Slug by siRNA in SW1116, and then determined the effect of Slug suppression on cell migration induced by TCM or CCL5. The results showed that Slug was effectively knocked down in SW1116 cells as supported by the expression of both mRNA and protein levels of Slug was dramatically downregulated (Figures 4a and b). Interestingly, knockdown of Slug significantly alleviated the promoting effect of TCM or CCL5 on SW1116 migration (Figure 4c). To illustrate a direct effect of CCL5 on Slug expression, we knocked down CCL5 expression in hMSCs by ccl5siRNA and examined the expression of Slug in SW1116 after TCM stimulation. Our result showed that ccl5 siRNA markedly decreased the TCM-induced upregulation of Slug in SW1116 cells (Figure 4d), suggesting that hMSC-induced upregulation of Slug in colon cancer cells is attributed to CCL5 secretion.

**Figure 5:**
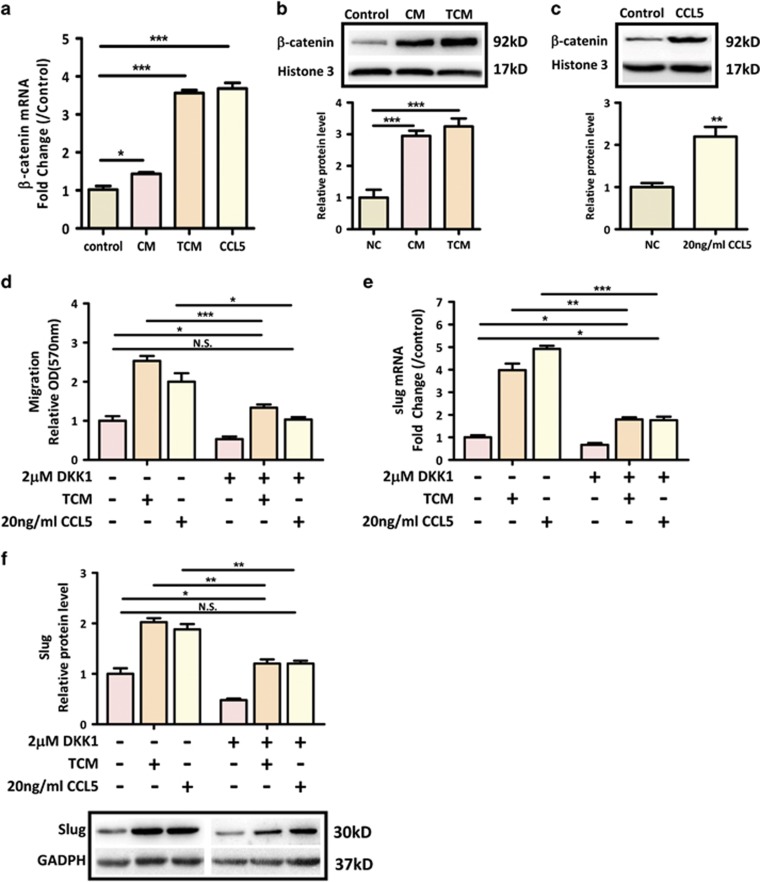
CCL5 activates *β*-catenin pathway. (**a**) The expression of *β*-catenin was determined by real-time PCR in SW1116 cells treated with CM/TCM or 20 ng/ml CCL5 for 24 hours. *P<0.05, ***P<0.001 *versus* control group; (**b**,**c**) The nuclear expression of *β*-catenin was determined by western blot in SW1116 cells treated with CM/TCM (**b**) or 20 ng/ml CCL5 (**c**) for 24 hours. **P<0.01, ***P<0.001 *versus* control group. Representative blots were shown in above; (**d**) The effect of Wnt/*β*-catenin inhibitor DKK1 on TCM- or CCL5-induced migration was determined by transwell assay. 2 *μ*M DKK1 was added into SW1116 cells and seeded in the upper chamber, TCM or 20 ng/ml CCL5 were added in the lower chamber. Quantification was presented as mean±S.D. from three independent experiments. *P<0.05, ***P<0.001; (**e**,**f**) 2 *μ*M DKK1 was added simultaneously with 20 ng/ml CCL5 or TCM into SW1116, the expression of Slug was determined by real-time PCR (**e**) or western blot (**f**). Data are presented as the means±S.D., *n*=3. *P<0.05, **P<0.01, ***P<0.001. Representative blot was shown in below **f**

Wnt/*β*-catenin pathway has been reported to regulate Slug transcription,^33^ and is critical for EMT process and colon cancer progression. Hence, it is plausible that Wnt/*β*-catenin pathway mediates the crosstalk between hMSCs and Slug-mediated EMT process in colon cancer cells. Interestingly, our real-time RT-PCR data revealed that exposure of SW1116 cells to either CM/TCM or CCL5 led to a dramatic increase in the expression of *β*-catenin (Figure 5a). Similar results were found in western blot analysis showing that the expression of nuclear *β*-catenin was significantly increased in SW1116 in response to TCM or CCL5 (Figures 5b and c). In addition, our luciferase assay showed that TCM treatment significantly activated *β*-catenin transcriptional activity in SW1116 cells (Supplementary Figure 3). To further investigate the functional impact of Wnt/*β*-catenin pathway on hMSC-mediated colon cancer progression, SW1116 was treated with DKK1, an antagonistic inhibitor of the Wnt/*β*-catenin signaling pathway. Then, the effects of TCM or CCL5 on cell migration and Slug expression were assessed in SW1116. Our result showed that inhibition of Wnt/*β*-catenin pathway significantly decreased TCM- or CCL5-mediated migration in SW1116 cells (Figure 5d). In corroboration with this result, DKK1 also dramatically abrogated the induction of Slug caused by TCM and CCL5 (Figures 5e and f). Altogether, these results indicate that CCL5/*β*-catenin/Slug pathway mediates the promotive effect of preactivated-hMSCs on colon cancer progression.

### hMSCs promote colon cancer development and EMT *in vivo*

**Figure 6:**
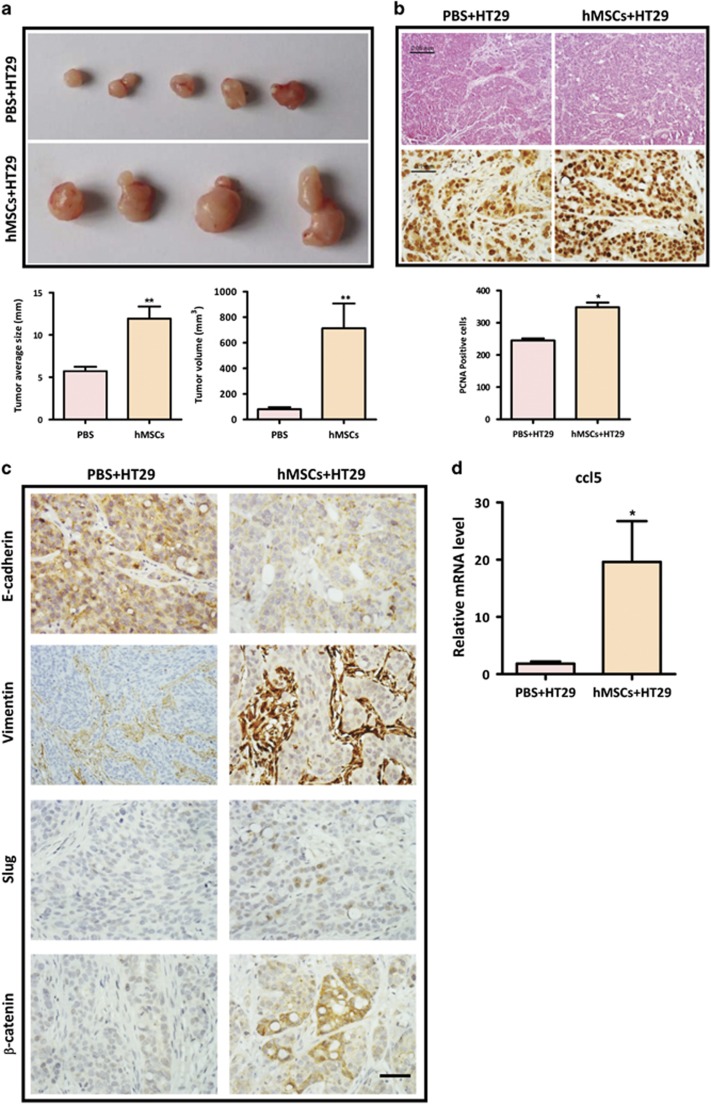
hMSCs promote CRC development and EMT *in vivo* BALB/C mice were subcutaneously injected with 1 × 10^7^ HT29 cells mixed with 1x10^7^ hMSCs (*n*=4) or alone (*n*=5). (**a**) The tumors were excised after 2 weeks, and tumor average size and tumor volume in xenograft model were measured. **P<0.01, *versus* PBS group; (**b**) H&E staining and immunohistochemical staining for PCNA (proliferating cell nuclear antigen) of xenograft tumors from either HT29 alone or co-injection group; quantification data are from at least five high fields. *P<0.05. Scale bar, 50 *μ*m and 100 *μ*m; (**c**) immunohistochemical staining for EMT markers (E-cadherin and Vimentin), *β*-catenin and Slug. Scale bar, 100 *μ*m; (**d**). The expression of CCL5 is increased in nude mice injected with HT29 and hMSCs. Tumor tissues were collected from mice injected with HT29 alone or HT29 with hMSCs. RNA was extracted and determined for expression of CCL5 by real-time PCR. Data are presented as the means±S.D. *n*=3 *P<0.05 *versus* control

Having established that hMSCs promote colon cancer cell EMT and progression *in vitro*, we further tested whether hMSCs promoted colon cancer development *in vivo*. Balb/C nude mice were subcutaneously injected with HT29 cells only or HT29 cells with hMSCs to establish a murine xenograft model of CRC. The primary tumor mass of tumor-bearing mice was monitored until killing. Our results showed that mice implanted with HT29 and hMSCs (*n*=4) exhibited a significantly increased tumor burden compared with mice (*n*=5) implanted with HT29 only (Figure 6a). To understand the effect of hMSCs on tumor growth, the subcutaneous tumor sections were stained for PCNA, which is a nuclear protein associated with proliferation at the experimental endpoint. As shown in Figure 6b, immunohistochemical analysis of xenograft tumors revealed that tumors with co-injection displayed higher frequency of mitotic figures. To evaluate the role of hMSCs in EMT process *in vivo*, we stained the tumor sections with epithelial marker E-cadherin and mesenchymal marker Vimentin. Our results showed that the expression of E-cadherin was significantly decreased whereas the expression of Vimentin was increased in HT29+hMSCs group (Figure 6c). Furthermore, in line with the *in vitro* data, tumors with co-injection exhibited a much enhanced expression of *β*-catenin and Slug compared with the tumors injected with HT29 only (Figure 6c). Of note, the expression of ccl5 mRNA was also significantly increased in tumors injected with both HT29 and hMSCs (Figure 6d). Thus, consistent with the in *vitro* results, our *in vivo* data supports the notion that hMSCs promote CRC EMT and development.

## Discussion

MSCs is one of the major components in the CRC stroma, which is directly involved in cancer development as demonstrated in mouse models.^6, 15, 16^ However, the exact role of MSCs in the development of CRC is not clear. In this study, we show that TNF-*α* preactivated-hMSCs exert a much stronger tumor-promoting effect than inactivated hMSCs via CCL5/*β*-catenin/Slug pathway in CRC development. Therefore, this study reveals a novel mechanism through which MSCs promote colon cancer progression, and emphasizes the importance of inflammatory cytokines/chemokines in the crosstalk between MSCs and cancer cells.

It has been well established that inflammation plays a critical role in every stage of tumor progression, and inflammatory cytokines in the tumor microenvironment are crucial in modulating the functions of various types of tumor stromal cells including MSCs.^34, 35^ In fact, as a master regulator of tumor-associated inflammation, TNF-*α* plays a major role in the enhancement of tumor progression by its modulation of multiple tumor-related cell types.^36, 37^ In the present study, we treated human BM-MSCs with TNF-*α* to mimic tumor-associated MSCs and investigated the influence of TNF-*α*-activated MSCs on CRC cell lines. We demonstrated that TNF-*α* preactivated hMSCs more significantly increased the cell growth and metastatic capacity of CRC cells than untreated hMSCs did, as demonstrated by *in vitro* MTT, migration and invasion assays (Figures 1a and 2c–e). Importantly, only preactivated-hMSCs could activate the key EMT transcription factors, but not untreated hMSCs (Figures 2a and b). In line with this result, co-incubation with TNF-*α*-pretreated hMSCs facilitated colon cancer cells to lose their epithelial characteristics and gain mesenchymal properties (Figure 1b). This shift is important since EMT is an integral component of CRC progression which confers motility and migration to cancer cells toward an invasive and metastatic phenotype.^38, 39^ Emerging evidence has indicated that the tumor microenvironment is a potent factor that may facilitate and even initiate EMT.^40^ On the other hand, Ren G *et al.,*^11^ showed that when BM-MSCs were treated with various inflammatory cytokines IFN*γ*, TNF*α*, IL-1, IL-6, and GM-CSF, only TNF-*α*-treated BM-MSCs displayed a profile of cytokine/chemokine production resembling that of tumor-associated MSCs. In support of these previous findings, the present study clearly shows that TNF-*α*-treated BM-MSCs promote colon cancer cell EMT and progression. It is plausible that during tumorigenesis, BM-MSCs are substantially recruited to the tumor microenvironment and are continuously exposed to the local inflammatory factors, such as TNF-*α*. This inflammatory milieu illustrates the BM-MSCs to fulfill distinctive features, such as the overexpression of cytokines/chemokines, which eventually activate EMT program in colon cancer cells.

The role of CCL5 and its receptors in cancer development has been recognized recently.^23^ CCL5/CCR axis has been reported to be over-activated in various cancers and involved in multiple steps of cancer progression, including proliferation, migration, invasion, angiogenesis, and metastatic colonization. Moreover, elevated CCL5 levels have been reported to be a biomarker for cancer and for predicting the prognosis and the development of therapeutic strategies.^41^ Regardless of these observations, the exact functions of CCL5/CCR axis in tumor biology are still unclear. We demonstrated in this study that TNF-*α*-pretreated hMSCs secreted high levels of CCL5 (Figure 3a), through which communicated with CRC cells and enhanced cancer EMT and progression. Ccl5 antisense treatment in hMSCs completely abolished the stimulatory effect of TCM on SW1116 migration (Figure 3b), supporting the notion that CCL5 secreted by primed-hMSCs is essential for the paracrine effect of hMSCs on colon cancer progression. While CCL5 can bind to CCR1, CCR3, and CCR5, we revealed that the paracrine CCL5-induced cell migration in colon cancer cells was mainly mediated by CCR1, given that CCR1 antagonist completely abolished the increased migration in colon cancer cells in response to CCL5 (Figure 3d). In addition, CCR1 antagonist dramatically ameliorated CCL5-induced upregulation of EMT markers (Figure 3e). Taken together, CCL5/CCR1 axis appears to be crucial in mediating a crosstalk between MSCs and cancer cells to induce colon cancer cell EMT and metastasis. Of note, CCL5 also concurs with the interaction between breast cancer cells and MSCs. It was reported that breast cancer cells stimulated CCL5 secretion by MSCs, and CCL5 in turn induced tumor cell migration and promotes invasion and metastasis.^42^ While human and mouse MSCs routinely express low levels of selected chemokines and receptors,^43, 44^ we show that TNF-*α*-pretreated hMSCs is sufficient to drive the paracrine CCL5-mediated activation of EMT program and metastasis in cancer cells.

One of the major findings in this study is that we have identified a novel signaling pathway mediating the paracrine effect of CCL5 on colon cancer cell progression. Our results show that TNF-*α*-pretreated hMSCs promote colon cancer EMT and metastasis via *β*-catenin/Slug pathway. Slug belongs to the Snail family and is a well-known EMT-inducing transcription factor/E-cadherin transcriptional repressor.^45^ Increasing evidence has revealed that Slug is elevated in a number of cancers and that its expression is correlated with invasiveness, metastasis, and poor prognosis. In colon cancer, Slug has been implicated in cancer cell EMT and tumor progression.^46, 47^ In this study, SW1116 cells treated with TCM or CCL5 dramatically increased Slug expression (Figures 4a and b). Selective inhibition of Slug by siRNA significantly reversed the effects of TCM or CCL5 on cell migration (Figure 4c). Furthermore, ccl5 siRNA treatment in hMSCs markedly alleviated the induction of Slug expression by TCM (Figure 4d). These results indicate that phenotypic transition of colon cancer cells by TNF-*α*-pretreated hMSCs involves CCL5-mediated upregulation of Slug. Canonical Wnt-mediated *β*-catenin activation induces various target genes that regulate cancer cell growth and metastasis. This pathway is particularly important in the intestine where mutations in genes involved in *β*-catenin degradation occur in over 90% of sporadic CRC.^48^ Thus, it is plausible that environmental signals play an important role in the malignant progression of CRC by activating Wnt/*β*-catenin pathway. For the first time, we demonstrated that CCL5 directly induced nuclear accumulation and activation of *β*-catenin in colon cancer cells (Figures 5a, b and Supplementary Figure 3). If the tumor-promoting effects of hMSCs are largely due to the CCL5/*β*-catenin pathway, the effects observed with TCM treatment should be reversed by Wnt/*β*-catenin suppression. To test this, we treated SW1116 cells with Wnt/*β*-catenin antagonist DKK1, and found that DKK1 dramatically reversed TCM-or CCL5-induced cell migration and upregulation of Slug expression in SW1116 cells (Figures 5c–e), indicating that upregulation of Wnt/*β*-catenin pathway is the major mechanism leading to the observed increased malignancies induced by hMSCs.

The tumor-promoting effects of hMSCs were demonstrated *in vivo* in a mouse model of CRC, as co-injection of HT29 with hMSCs markedly increased tumor burden (Figures 6a and b). Immunohistochemical staining revealed the manifestation of EMT in the co-injection group, as the expression of E-cadherin was significantly decreased whereas the expression of Vimentin was increased in the mice co-injected with hMSCs (Figure 6c). Moreover, consistent with the *in vitro* results, the ccl5/*β*-catenin/Slug pathway was over-activated in the tumors co-injected with hMSCs (Figures 6c and d). Collectively, these results suggest that CCL5/CCR1/*β*-catenin/Slug pathway is responsible for the tumor-promoting effects of TNF-*α*-activated hMSCs on the development of colon cancer. Furthermore, our findings provide important insights into the role of MSCs in promoting cancer progression, as well as the importance of inflammation in this effect. Strategies that target MSCs-cancer cells crosstalk should provide a novel avenue of cancer therapy. On the other hand, the modulation of MSCs by resident inflammation may also implicate in other chronic diseases, such as inflammatory bowel disease.

## Materials and methods

### Cell lines

Human bone marrow-derived MSCs were purchased from ATCC (PCS-500-012; Manassas, VA, USA), and cultured in *α*-modified Eagle’s medium (MEM) supplemented with 10% FBS and 1% PS in an atmosphere of 5% CO_2_ at 37 °C. hMSCs were defined by chondrogenic, osteogenic, and adipogenic differentiation *in vitro* according to standard conditions reported previously.^49^ Human colon cancer cell lines HT29, SW1116, Caco2, Lovo and normal rat intestinal epithelial cell line IEC-18 were purchased from ATCC or Shanghai Institutes for Biological Sciences (Shanghai, China) and cultured in MEM or RMPI-1640 supplemented with 10% fetal bovine serum, 1% penicillin, 1% streptomycin at 37 °C in 5% CO_2_. All cell culture medium and antibiotics were obtained from Life Technologies (Carlsbad, CA, USA).

### Collection of conditional media and coculture

hMSCs (p4-p10) were seeded in 75 cm^2^ flask and cultured to 80% confluence. After washed with PBS for three times, serum-free medium with or without 10 ng/ml TNF-*α* was added. After 24 h, medium without TNF-*α* pretreatment was collected, filtered, and stored at −80 °C as CM. Alternatively, cells with TNF-*α* pretreatment were washed with PBS for three times, and cultured for another 24 h in fresh serum-free medium. After 24 h, medium was collected, filtered, and stored at −80 °C as TCM. For coculture experiment, 0.4 *μ*m Transwell insert (Corning, New York, USA) was applied. MSCs cultured in serum-free media with or without TNF-*α* pretreatment were seeded in the upper chamber, while tumor cells were seeded in the lower chamber. As the control for CM or TCM treatment, serum-free *α*-MEM was used for culturing colon cancer cell lines.

### siRNA knockdown

Ccl5 or Slug knockdown was achieved by transfecting hMSCs or SW11116 with a final concentration of 10 pmol ccl5 or Slug siRNAs. Scrambled control siRNA was used as control (Ambion, Life Technologies) using 5 *μ*l Lipofectamine 2000 (Life Technologies). Three days after transfection, cells were harvested for western blot or real-time PCR analysis or functional assays.

### Proliferation assay

MTT assay was applied to assess cell proliferation activity. Colon cancer cell lines were seeded into 96-well plates and allowed to adhere overnight. After addition of CM or TCM for different time points, MTT reagent of 20 *μ*l (5 mg/ml, Sigma-Aldrich, St. Louis, MO, USA) was added into each well to terminate the experiments. OD value at 570/630 nm was recorded in each well using a microliter plate reader.

### Transwell migration assay

Transwell assays were performed as previously described.^50^ In brief, cells were trypsinized and seeded into the upper chamber of transwell (6.5 mm diameter, 8 *μ*m pore size, Corning). 2.5 × 10^4^ colon cancer cells were seeded in 200 *μ*l serum-free media in each well. In the lower chamber, serum containing media, CM or growth factors were added as indicated in different experiments. For negative control, only basal media were added into lower chamber. Following 24 h incubation, cells remaining on the upper surface of the filter were removed with a cotton swab. Cells that had migrated to the lower surface were fixed with 4% PFA for 10 min and stained with 0.5% crystal violet for another 10 min. The average numbers of migrated cells were determined by counting the cells in three or more random fields under microscope. In some experiment, OD value at 570 nm was recorded in each well using a microliter plate reader. To determine the role of CCL5/CCR1/*β*-catenin signaling in CRC progression, SW1116 were seeded into the upper chambers of transwell together with CCL5 receptor antagonists BX471 (Sigma, sml0020) or Wnt inhibitor DKK1 (R&D, 5439-DK) and evaluated for migratory ability.

### Cell scratch assay

To test cell migration ability, colon cancer cells were seeded in six-well plates. When the cells reached ~90–100% confluence as a monolayer, 200 *μ*l pipette tips were used to scratch on the surface of cell culture dish to generate a gap. Then, cells were washed and re-incubated in serum-free media with or without CM/TCM. Cell migration was monitored under live image microscope every 1 h. Migrated areas were calculated with Photoshop software.

### Spheroid invasion assay

Spheroid invasion was measured by placing HT29 spheroids in six-well plates as described in previous study.^51^ In brief, cells were trypsinized and diluted into 2.5 × 10^6^ cells/ml. Ten microliter drops cell suspension was deposited onto the lid of culture dish. Then, the lid was inverted onto the PBS-filled bottom chamber and incubated at 37 °C. After cultured for 4–7 days, formed spheroids were picked out for further analysis. We typically analyze images using ImageJ software.

### Enzyme-linked Immunosorbent Assay

Conditioned medium (CM or TCM) from MSC culture was collected by centrifuging at 10 000 × *g* for 10 min to remove cell debris. The level of CCL5 was measured using the ELISA kit obtained from R&D Systems (Minneapolis, MN, USA) according to the manufacturer’s instructions.

### Dual-luciferase Report assay

Sub-confluent SW1116 cells were seeded in 24-well plate and transfected with 0.5 *μ*g *β*-catenin/TCF4 luciferase reporter (pTop-luc; Millipore, Billerica, MA, USA) per well with Lipofectamine 2000 (Invitrogen, Life Technologies). After incubation for 12 h, cells were treated with TCM or 0.1% DMSO. 72 h later, cells were lysed and subjected to luciferase assays using Dual-Luciferase Reporter Assay System (Promega, cat# E1910) and the LB 96 V MicroLumat Plus (EG&G Berthold Technologies).

### Quantitative real-time RT-PCR (qRT-PCR)

Total RNA was isolated using TRIZOL Reagent (Invitrogen, Life Technologies), 1–5 *μ*g total RNA were used for reverse transcription, first-strand complementary DNA synthesis was performed using oligd(T)_18_ and M-MLV enzyme (Promega, Madison, WI, USA). The levels of mRNA were measured by real-time PCR (Applied Biosystems, 7500, USA) using SYBR Green Master Mix (Applied Biosystems). Total amount of mRNA was normalized to endogenous gapdh mRNA. The sequences of the primers were shown in Supplementary Table 1.

### Immuohistochemical Staining

Tumor tissues were fixed in 4% paraformaldehyde in PBS at 4 °C for 24 h and then embedded in paraffin. Tissues were cut into 4*μ*m sections and de-paraffined three times in xylene and rehydrated in gradient alcohols. Endogenous peroxidase activity was quenched with 3% H_2_O_2_ in methanol for 10 min, and sections were washed in PBS. The de-paraffinized sections were heated by microwave and boiled for 20 min in 10 mM citrate buffer (pH 6.0) for antigen retrieval. Sections were blocked with horst serum for 30 min and then incubated with respective primary antibodies at 4 °C overnight and horseradishperoxidase-conjugated secondary antibodies (rabbit, mouse, or goat, Gene Tech) at room temperature for 30 min. Sections were developed with diaminobenzidine and counterstained with hematoxylin & eosin using standard protocols. Antibodies used include PCNA (PC10, CST-2586, 1:1000), *β*-Catenin (D10A8, CST-8480, 1:100), Slug (C19G7, CST-9585, 1:400), E-Cadherin (24E10, CST-3195 1:200), and Vimentin (D21H3, CST-5741, 1:100).

### Western blot analysis

Protein (20–40 *μ*g) was separated on a 10% SDS–PAGE gel and transferred onto a nitrocellulose membrane. After blocking with 5% milk for 1 h, the membranes were incubated overnight at 4 °C with primary antibody, then washed three times with Tris Buffered Saline-Tween 20 followed by another hour of incubation with the corresponding secondary antibody at room temperature. Finally, the blot was subjected to chemiluminescent detection with ECLTM Prime Detection Reagent (Amersham GE Care).

### Xenograft mice model

Tumorigenicity was investigated by tumor xenograft experiments. The athymic female nude mice of 6–8 weeks old were purchased from Shanghai Laboratory Animal Center (Shanghai, China) and maintained in filter-topped units. All animal experiments were conducted in accordance with the guidelines and regulations on animal experimentation of the Chinese University of Hong Kong and approved by the Animal Ethnics Committee of the University. 1 × 10^7^ HT29 cells and 1 × 10^7^ hMSCs were suspended in 100 *μ*l PBS and injected subcutaneously. 1 × 10^7^ HT29 cells with PBS were injected in the contralateral side as control. Tumor formation in nude mice was monitored over about 2 week period and tumor volumes were measured.

### Statistical analysis

Values were expressed as mean±S.E.M. and analyzed with the Student’s *t*-test using GraphPad prism 5 software (GraphPad Software, San Diego, CA, USA). Differences with *P* values <0.05 were considered statistically significant.

## References

[bib1] Siegel R, Desantis C, Jemal A. Colorectal cancer statistics, 2014. CA Cancer J Clin 2014; 64: 104–117. 24639052 10.3322/caac.21220

[bib2] Yokota T, Kunii Y, Kagami M, Yamada Y, Takahashi M, Kikuchi S et al. Metastatic breast carcinoma masquerading as primary colon cancer. Am J Gastroenterol 2000; 95: 3014–3016. 11051411 10.1111/j.1572-0241.2000.03238.x

[bib3] Pizzini S, Bisognin A, Mandruzzato S, Biasiolo M, Facciolli A, Perilli L et al. Impact of microRNAs on regulatory networks and pathways in human colorectal carcinogenesis and development of metastasis. BMC Genomics 2013; 14: 589. 23987127 10.1186/1471-2164-14-589 PMC3766699

[bib4] Tse JC, Kalluri R. Mechanisms of metastasis: epithelial-to-mesenchymal transition and contribution of tumor microenvironment. J Cell Biochem 2007; 101: 816–829. 17243120 10.1002/jcb.21215

[bib5] Whiteside TL. The tumor microenvironment and its role in promoting tumor growth. Oncogene 2008; 27: 5904–5912. 18836471 10.1038/onc.2008.271 PMC3689267

[bib6] Kalluri R, Zeisberg M. Fibroblasts in cancer. Nat Rev Cancer 2006; 6: 392–401. 16572188 10.1038/nrc1877

[bib7] Mishra PJ, Mishra PJ, Humeniuk R, Medina DJ, Alexe G, Mesirov JP et al. Carcinoma-associated fibroblast-like differentiation of human mesenchymal stem cells. Cancer Res 2008; 68: 4331–4339. 18519693 10.1158/0008-5472.CAN-08-0943 PMC2725025

[bib8] Mishra PJ, Merlino G. A traitor in our midst: mesenchymal stem cells contribute to tumor progression and metastasis. Future Oncol 2008; 4: 745–749. 19086838 10.2217/14796694.4.6.745

[bib9] Quante M, Tu SP, Tomita H, Gonda T, Wang SS, Takashi S et al. Bone marrow-derived myofibroblasts contribute to the mesenchymal stem cell niche and promote tumor growth. Cancer Cell 2011; 19: 257–272. 21316604 10.1016/j.ccr.2011.01.020 PMC3060401

[bib10] Studeny M, Marini FC, Dembinski JL, Zompetta C, Cabreira-Hansen M, Bekele BN et al. Mesenchymal stem cells: potential precursors for tumor stroma and targeted-delivery vehicles for anticancer agents. J Natl Cancer Inst 2004; 96: 1593–1603. 15523088 10.1093/jnci/djh299

[bib11] Ren G, Zhao X, Wang Y, Zhang X, Chen X, Xu C et al. CCR2-dependent recruitment of macrophages by tumor-educated mesenchymal stromal cells promotes tumor development and is mimicked by TNFalpha. Cell Stem Cell 2012; 11: 812–824. 23168163 10.1016/j.stem.2012.08.013 PMC3518598

[bib12] Tang RJ, Shen SN, Zhao XY, Nie YZ, Xu YJ, Ren J et al. Mesenchymal stem cells-regulated Treg cells suppress colitis-associated colorectal cancer. Stem Cell Res Ther 2015; 6: 71. 25889203 10.1186/s13287-015-0055-8 PMC4414289

[bib13] Nasuno M, Arimura Y, Nagaishi K, Isshiki H, Onodera K, Nakagaki S et al. Mesenchymal stem cells cancel azoxymethane-induced tumor initiation. Stem Cells 2014; 32: 913–925. 24715689 10.1002/stem.1594

[bib14] Tsai KS, Yang SH, Lei YP, Tsai CC, Chen HW, Hsu CY et al. Mesenchymal stem cells promote formation of colorectal tumors in mice. Gastroenterology 2011; 141: 1046–1056. 21699785 10.1053/j.gastro.2011.05.045

[bib15] De Boeck A, Pauwels P, Hensen K, Rummens JL, Westbroek W, Hendrix A et al. Bone marrow-derived mesenchymal stem cells promote colorectal cancer progression through paracrine neuregulin 1/HER3 signalling. Gut 2013; 62: 550–560. 22535374 10.1136/gutjnl-2011-301393

[bib16] Mele V, Muraro MG, Calabrese D, Pfaff D, Amatruda N, Amicarella F et al. Mesenchymal stromal cells induce epithelial-to-mesenchymal transition in human colorectal cancer cells through the expression of surface-bound TGF-beta. Int J Cancer 2014; 134: 2583–2594. 24214914 10.1002/ijc.28598 PMC4338537

[bib17] Wu XB, Liu Y, Wang GH, Xu X, Cai Y, Wang HY et al. Mesenchymal stem cells promote colorectal cancer progression through AMPK/mTOR-mediated NF-kappaB activation. Sci Rep 2016; 6: 21420. 26892992 10.1038/srep21420 PMC4759824

[bib18] Lin SJ, Chang KP, Hsu CW, Chi LM, Chien KY, Liang Y et al. Low-molecular-mass secretome profiling identifies C-C motif chemokine 5 as a potential plasma biomarker and therapeutic target for nasopharyngeal carcinoma. J Proteomics 2013; 94: 186–201. 24080422 10.1016/j.jprot.2013.09.013

[bib19] Homey B, Muller A, Zlotnik A. Chemokines: agents for the immunotherapy of cancer? Nat Rev Immunol 2002; 2: 175–184. 11913068 10.1038/nri748

[bib20] Soria G, Ben-Baruch A. The inflammatory chemokines CCL2 and CCL5 in breast cancer. Cancer Lett 2008; 267: 271–285. 18439751 10.1016/j.canlet.2008.03.018

[bib21] Mrowietz U, Schwenk U, Maune S, Bartels J, Kupper M, Fichtner I et al. The chemokine RANTES is secreted by human melanoma cells and is associated with enhanced tumour formation in nude mice. Br J Cancer 1999; 79: 1025–1031. 10098731 10.1038/sj.bjc.6690164 PMC2362228

[bib22] Sugasawa H, Ichikura T, Tsujimoto H, Kinoshita M, Morita D, Ono S et al. Prognostic significance of expression of CCL5/RANTES receptors in patients with gastric cancer. J Surg Oncol 2008; 97: 445–450. 18297689 10.1002/jso.20984

[bib23] Aldinucci D, Colombatti A. The inflammatory chemokine CCL5 and cancer progression. Mediators inflamm 2014; 2014: 292376. 24523569 10.1155/2014/292376 PMC3910068

[bib24] Cambien B, Richard-Fiardo P, Karimdjee BF, Martini V, Ferrua B, Pitard B et al. CCL5 neutralization restricts cancer growth and potentiates the targeting of PDGFRbeta in colorectal carcinoma. PLoS ONE 2011; 6: e28842. 22205974 10.1371/journal.pone.0028842 PMC3243667

[bib25] Kan JY, Wu DC, Yu FJ, Wu CY, Ho YW, Chiu YJ et al. Chemokine (C-C Motif) ligand 5 is involved in tumor-associated dendritic cell-mediated colon cancer progression through non-coding RNA MALAT-1. J Cell Physiol 2015; 230: 1883–1894. 25546229 10.1002/jcp.24918

[bib26] Levina V, Su Y, Nolen B, Liu X, Gordin Y, Lee M et al. Chemotherapeutic drugs and human tumor cells cytokine network. Int J Cancer 2008; 123: 2031–2040. 18697197 10.1002/ijc.23732 PMC2574811

[bib27] Chang LY, Lin YC, Mahalingam J, Huang CT, Chen TW, Kang CW et al. Tumor-derived chemokine CCL5 enhances TGF-beta-mediated killing of CD8(+) T cells in colon cancer by T-regulatory cells. Cancer Res 2012; 72: 1092–1102. 22282655 10.1158/0008-5472.CAN-11-2493

[bib28] Zhang Y, Lv D, Kim HJ, Kurt RA, Bu W, Li Y et al. A novel role of hematopoietic CCL5 in promoting triple-negative mammary tumor progression by regulating generation of myeloid-derived suppressor cells. Cell Res 2013; 23: 394–408. 23266888 10.1038/cr.2012.178 PMC3587709

[bib29] Kauts ML, Pihelgas S, Orro K, Neuman T, Piirsoo A. CCL5/CCR1 axis regulates multipotency of human adipose tissue derived stromal cells. Stem Cell Res 2013; 10: 166–178. 23276697 10.1016/j.scr.2012.11.004

[bib30] Lu L, Zhang X, Zhang M, Zhang H, Liao L, Yang T et al. RANTES and SDF-1 are keys in cell-based therapy of TMJ osteoarthritis. J Dent Res 2015; 94: 1601–1609. 26377571 10.1177/0022034515604621

[bib31] Wise AF, Williams TM, Kiewiet MB, Payne NL, Siatskas C, Samuel CS et al. Human mesenchymal stem cells alter macrophage phenotype and promote regeneration via homing to the kidney following ischemia-reperfusion injury. Am J Physiol Renal Physiol 2014; 306: F1222–F1235. 24623144 10.1152/ajprenal.00675.2013

[bib32] Findlay VJ, Wang C, Watson DK, Camp ER. Epithelial-to-mesenchymal transition and the cancer stem cell phenotype: insights from cancer biology with therapeutic implications for colorectal cancer. Cancer Gene Ther 2014; 21: 181–187. 24787239 10.1038/cgt.2014.15 PMC4041800

[bib33] Sakai D, Tanaka Y, Endo Y, Osumi N, Okamoto H, Wakamatsu Y. Regulation of Slug transcription in embryonic ectoderm by beta-catenin-Lef/Tcf and BMP-Smad signaling. Dev Growth Differ 2005; 47: 471–482. 16179074 10.1111/j.1440-169X.2005.00821.x

[bib34] Grivennikov SI, Greten FR, Karin M. Immunity, inflammation, and cancer. Cell 2010; 140: 883–899. 20303878 10.1016/j.cell.2010.01.025 PMC2866629

[bib35] Grivennikov SI, Karin M. Inflammation and oncogenesis: a vicious connection. Curr Opin Genet Dev 2010; 20: 65–71. 20036794 10.1016/j.gde.2009.11.004 PMC2821983

[bib36] Aran D, Lasry A, Zinger A, Biton M, Pikarsky E, Hellman A et al. Widespread parainflammation in human cancer. Genome Biol 2016; 17: 145. 27386949 10.1186/s13059-016-0995-z PMC4937599

[bib37] Lasry A, Zinger A, Ben-Neriah Y. Inflammatory networks underlying colorectal cancer. Nat Immunol 2016; 17: 230–240. 26882261 10.1038/ni.3384

[bib38] Bates RC, Mercurio AM. The epithelial-mesenchymal transition (EMT) and colorectal cancer progression. Cancer Biol Ther 2005; 4: 365–370. 15846061 10.4161/cbt.4.4.1655

[bib39] Bates RC, Pursell BM, Mercurio AM. Epithelial-mesenchymal transition and colorectal cancer: gaining insights into tumor progression using LIM 1863 cells. Cells Tissues Organs 2007; 185: 29–39. 17587805 10.1159/000101300

[bib40] Gout S, Huot J. Role of cancer microenvironment in metastasis: focus on colon cancer. Cancer Microenviron. 2008; 1: 69–83. 19308686 10.1007/s12307-008-0007-2 PMC2654352

[bib41] Moran CJ, Arenberg DA, Huang CC, Giordano TJ, Thomas DG, Misek DE et al. RANTES expression is a predictor of survival in stage I lung adenocarcinoma. Clin Cancer Res 2002; 8: 3803–3812. 12473593

[bib42] Karnoub AE, Dash AB, Vo AP, Sullivan A, Brooks MW, Bell GW et al. Mesenchymal stem cells within tumour stroma promote breast cancer metastasis. Nature 2007; 449: 557–563. 17914389 10.1038/nature06188

[bib43] Honczarenko M, Le Y, Swierkowski M, Ghiran I, Glodek AM, Silberstein LE. Human bone marrow stromal cells express a distinct set of biologically functional chemokine receptors. Stem Cells 2006; 24: 1030–1041. 16253981 10.1634/stemcells.2005-0319

[bib44] Ringe J, Strassburg S, Neumann K, Endres M, Notter M, Burmester GR et al. Towards *in situ* tissue repair: human mesenchymal stem cells express chemokine receptors CXCR1, CXCR2 and CCR2, and migrate upon stimulation with CXCL8 but not CCL2. J Cell Biochem 2007; 101: 135–146. 17295203 10.1002/jcb.21172

[bib45] Peinado H, Olmeda D, Cano A. Snail, Zeb and bHLH factors in tumour progression: an alliance against the epithelial phenotype? Nat Rev Cancer 2007; 7: 415–428. 17508028 10.1038/nrc2131

[bib46] Li Y, Zhao Z, Xu C, Zhou Z, Zhu Z, You T. HMGA2 induces transcription factor Slug expression to promote epithelial-to-mesenchymal transition and contributes to colon cancer progression. Cancer Lett 2014; 355: 130–140. 25218351 10.1016/j.canlet.2014.09.007

[bib47] Yao C, Su L, Shan J, Zhu C, Liu L, Liu C et al. IGF/STAT3/NANOG/Slug signaling axis simultaneously controls epithelial-mesenchymal transition and stemness maintenance in colorectal cancer. Stem Cells 2016; 34: 820–831. 26840943 10.1002/stem.2320

[bib48] Clevers H, Nusse R. Wnt/beta-catenin signaling and disease. Cell 2012; 149: 1192–1205. 22682243 10.1016/j.cell.2012.05.012

[bib49] Liu Y, Jiang X, Zhang X, Chen R, Sun T, Fok KL et al. Dedifferentiation-reprogrammed mesenchymal stem cells with improved therapeutic potential. Stem Cells 2011; 29: 2077–2089. 22052697 10.1002/stem.764

[bib50] Shang YC, Wang SH, Xiong F, Zhao CP, Peng FN, Feng SW et al. Wnt3a signaling promotes proliferation, myogenic differentiation, and migration of rat bone marrow mesenchymal stem cells. Acta Pharmacol Sin 2007; 28: 1761–1774. 17959027 10.1111/j.1745-7254.2007.00671.x

[bib51] Foty R. A simple hanging drop cell culture protocol for generation of 3D spheroids. J Vis Exp 2011: 51. 10.3791/2720 PMC3197119 21587162

