# Supplementary figures and images for: Human MSCs promotes colorectal cancer epithelial–mesenchymal transition and progression via CCL5/β-catenin/Slug pathway
Source: Cell Death Dis. 2017 May 25;8(5):e2819–. doi: 10.1038/cddis.2017.138 (PMC5520690; doi:10.1038/cddis.2017.138)

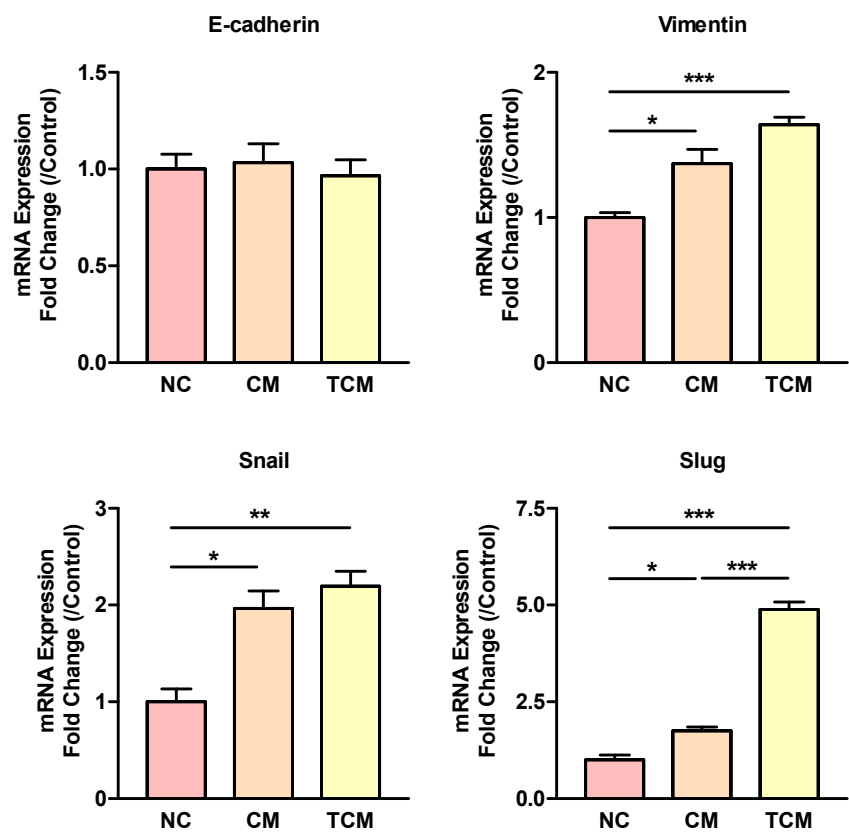

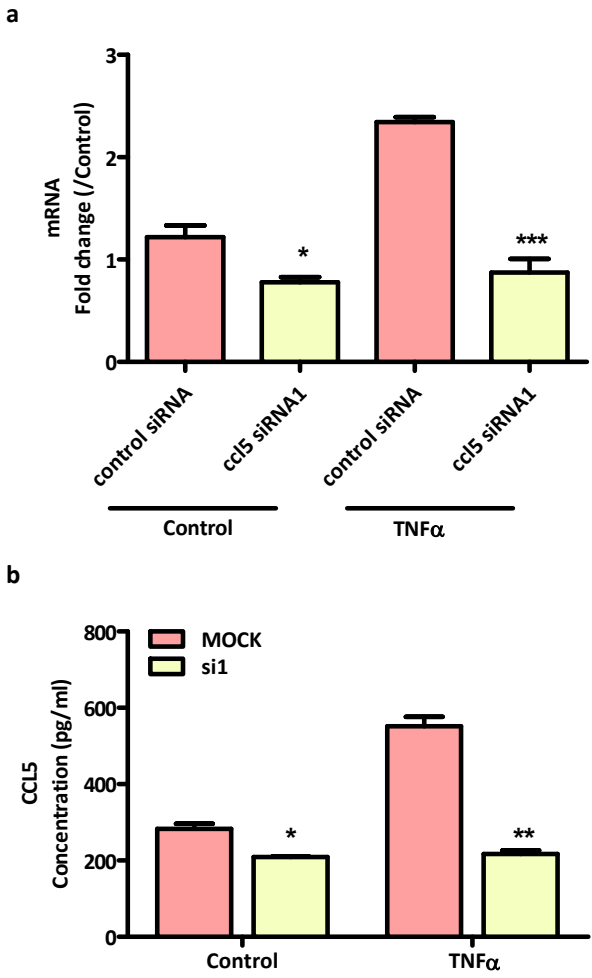

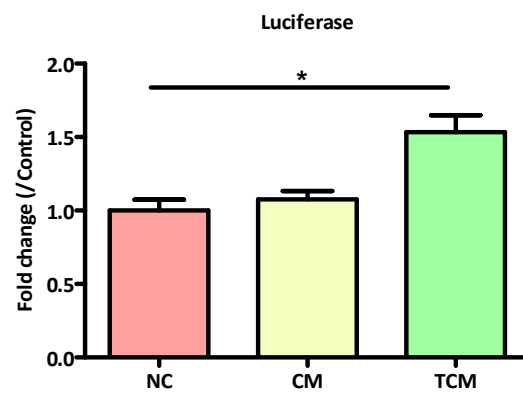

Supplement: Supplementary Figures [file cddis2017138x1.pdf]
