# Supplementary material for: Human MSCs promotes colorectal cancer epithelial–mesenchymal transition and progression via CCL5/β-catenin/Slug pathway
Source: Cell Death Dis. 2017 May 25;8(5):e2819–. doi: 10.1038/cddis.2017.138 (PMC5520690; doi:10.1038/cddis.2017.138)
Supplement: Supplementary Table 1 [file cddis2017138x2.pdf]

Supplementary Table 1: Primers used for the experiment				
	**Forward (5' - 3')**	**Reverse (5' - 3')**	**Expected size**	**Accession no.**
h-CCL5-rt2	GCTGCTTTGCCTACATT	CATTTCTTCTCTGGGTTG	142 bp	NM_002985.2
h-CCR1	CACCAATCAGTGTGAGCAGAG	AGCAGAGAGCTCATGTTCTCCT	253bp	NM_001295.2
h-CCR3	TCGTTCTCCCTCTGCTCG	AGATGCTTGCTCCGCTCA	214 bp	NM_001837.3
h-CCR5	AGGGCTGTGAGGCTTATC	GTTTGGCAATGTGCTTTT	262 bp	NM_000579.3
h-Slug (SNAI2)	TGACCTGTCTGCAAATGCTC	TCGGACCCACACATTACCTT	145 bp	NM_003068.4
h-Snai1	TTTACCTTCCAGCAGCCCTA	CCCACTGTCCTCATCTGACA	207bp	NM_005985.3
h-Ecadherin (CDH1)	GCCGAGAGCTACACGTTCAC	GTCGAGGGAAAAATAGGCTG	117 bp	NM_004360.4
h-Vimentin	GAGAACTTTGCCGTTGAAGC	TCCAGCAGCTTCCTGTAGGT	170 bp	NM_003380.3
h-ZEB1	CAGTCAGCTGCATCTGTAACAC	CCAGGTGTAAGCGCAGAAAG	145 bp	NM_030751.5
h-bcatenin (CTNNB1)	CGCTGGATTTTCAAAACAGT	CTGAGGAGCAGCTTCAGTCC	130 bp	NM_001904.3
h-GAPDH	AGGGTCATCATCTCTGCC	CCATCACGCCACAGTTTC	245 bp	NM_002046
