# Supplementary material for: Human MSCs promotes colorectal cancer epithelial–mesenchymal transition and progression via CCL5/β-catenin/Slug pathway
Source: Cell Death Dis. 2017 May 25;8(5):e2819–. doi: 10.1038/cddis.2017.138 (PMC5520690; doi:10.1038/cddis.2017.138)
Supplement: Supplementary Figures and Table Legends [file cddis2017138x3.docx]

**Supplementary figure legends:**

**Supplementary Figure 1. hMSCs promote EMT markers in SW1116.** After incubation with CM or TCM, the expression levels of EMT-related genes in SW1116 were evaluated by quantitative PCR. Data are presented as the means ± S.D. n = 3. *p<0.05, **p<0.01, ***p<0.001.

**Supplementary Figure 2**. **The expression of CCL5 after ccl5 siRNA treatment in hMSCs**

hMSCs were transfected with control or ccl5 siRNAs for 24 hours, and then treated with TNF-α for 24 hours. The cells were collected for further analysis. a. The mRNA expression was determined by Real-time PCR. Data are presented as the means ± S.D. n = 3. *p<0.05, **p<0.01, ***p<0.001; **b.** The condition media was collected and determined for CCL5 secretion by ELISA. Data are presented as the means ± S.D., n = 3. *p<0.05, **p<0.01.

**Supplementary Figure 3**. **Activated hMSCs induce β-catenin transcriptional activity**

Luciferase assay was performed to test the transcriptional activity of β-catenin after treatment with CM and TCM in SW1116 cells. Data are presented as the means ± S.D *p<0.05 vs. control.

**Supplementary Table 1: Primers used for the experiment.**
